# Supplementary material for: A genome-wide association study identifies 41 loci associated with eicosanoid levels
Source: Commun Biol. 2023 Jul 31;6:792. doi: 10.1038/s42003-023-05159-5 (PMC10390489; doi:10.1038/s42003-023-05159-5)
Supplement: Supplementary file 2 — Supplementary Information [file 42003_2023_5159_MOESM2_ESM.pdf]

Supplementary Figures

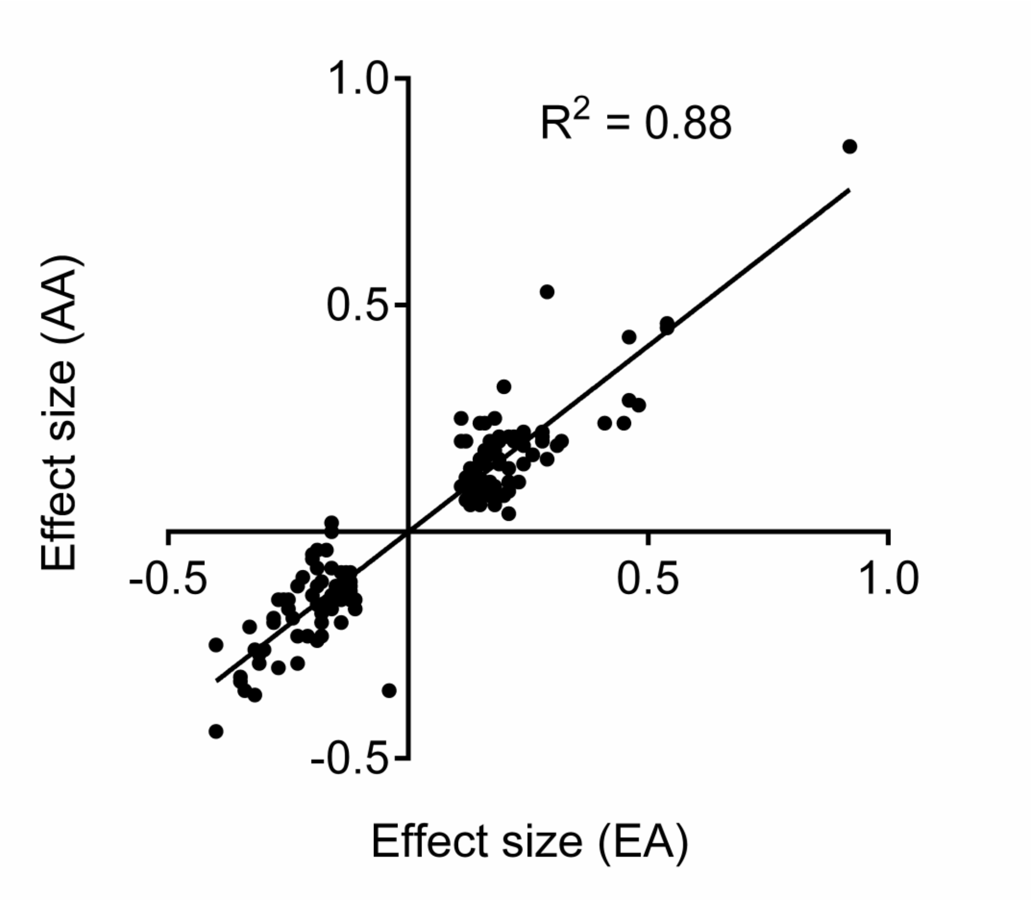

Supplementary Figure 1. Comparison of effect sizes at significant loci by ancestry. Scatter plot of effect sizes of significant eicosanoid GWAS loci in EA (x-axis) versus AA (y-axis) cohorts.

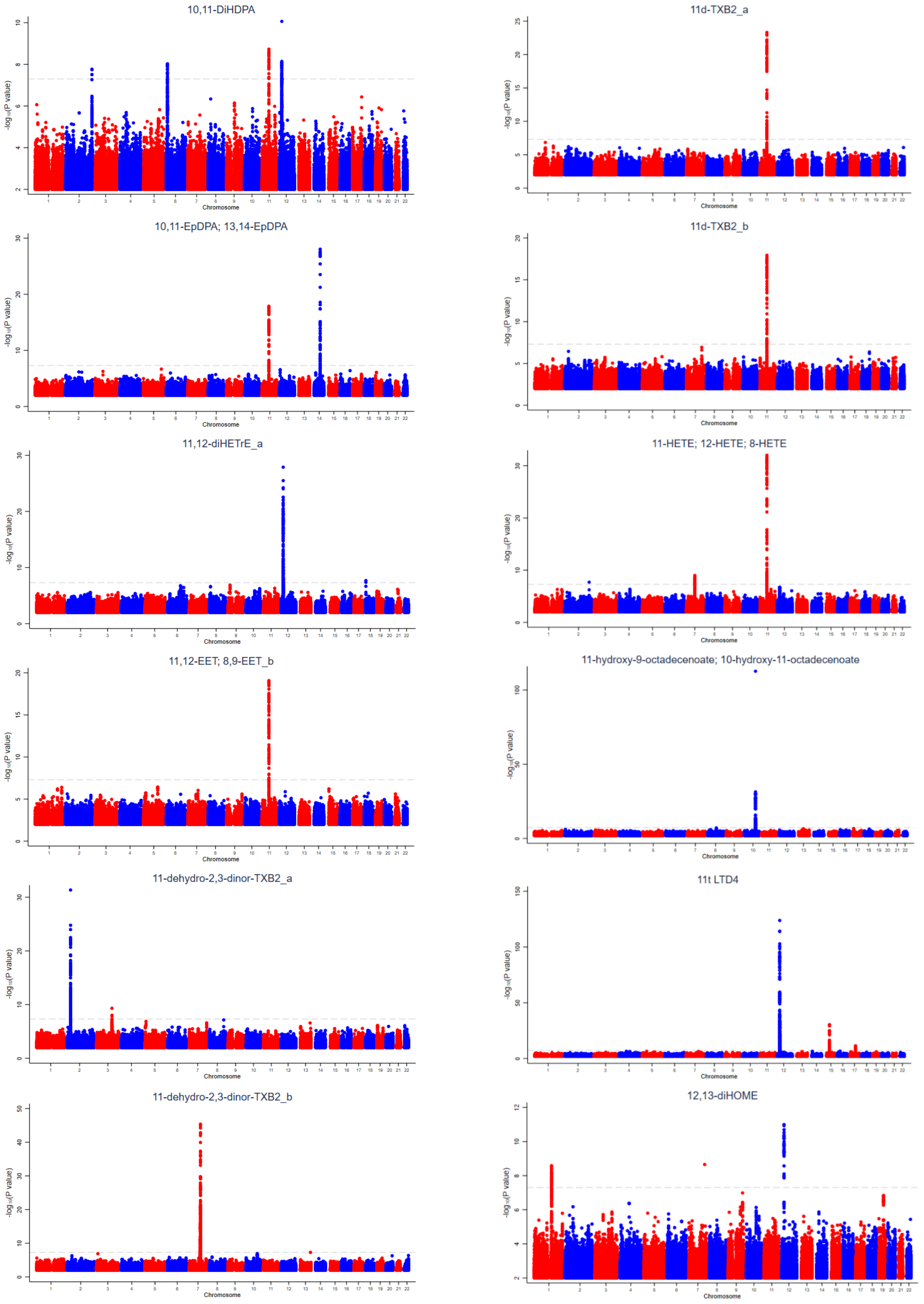

Supplementary Figure 2. Significant genome-wide eicosanoid associations (1-12). Manhattan plots for eicosanoids with at least one significant association, with association signals (-log10 of P-value) on the y-axis versus SNPs according to their position in the genome on the x-axis. Horizontal dashed lines represent Bonferroni adjusted significance threshold ($P < 2.24 \times 10^{-10}$).

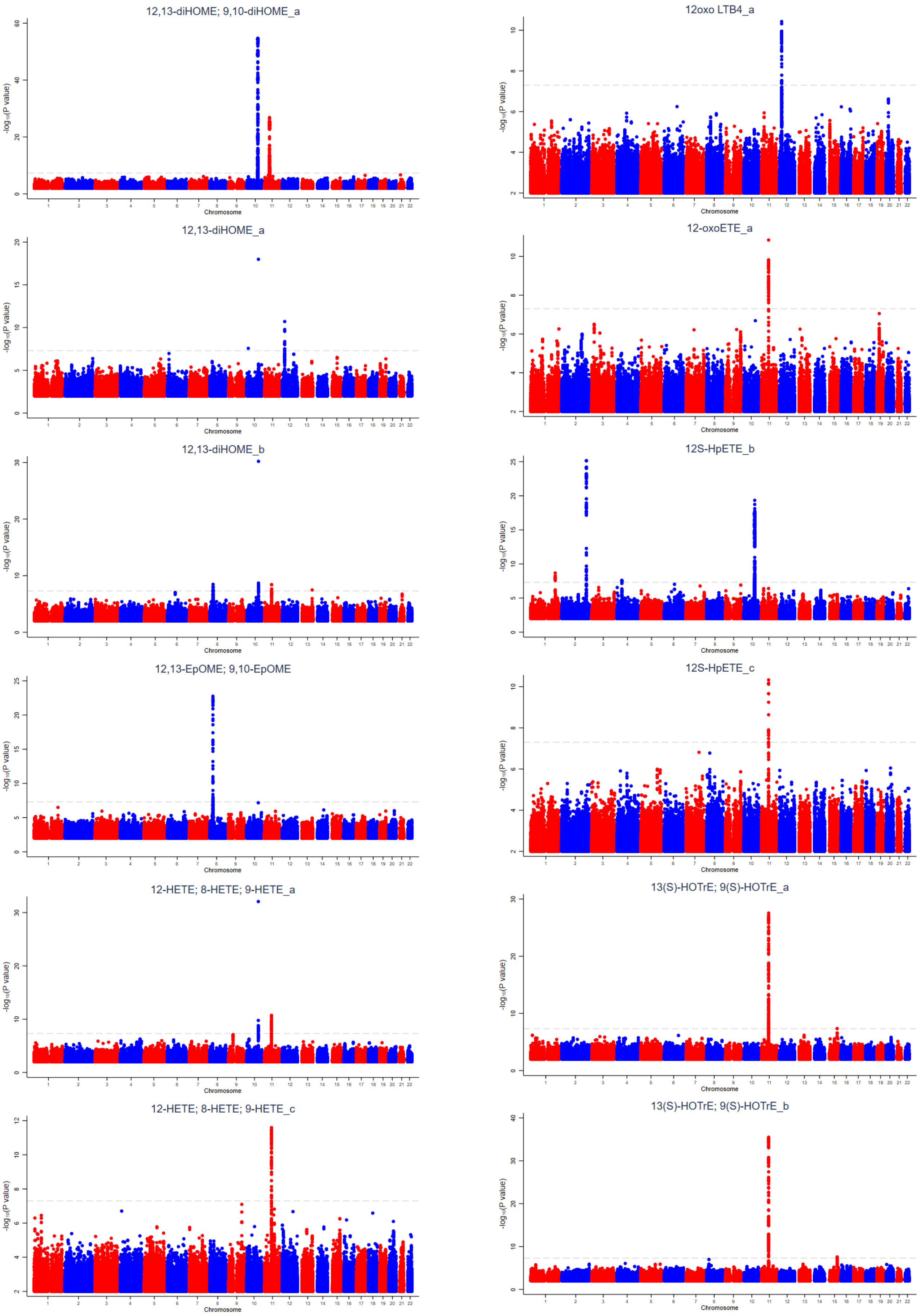

Supplementary Figure 3. Significant genome-wide eicosanoid associations (13-24). Manhattan plots for eicosanoids with at least one significant association, with association signals (-log10 of P-value) on the y-axis versus SNPs according to their position in the genome on the x-axis. Horizontal dashed lines represent Bonferroni adjusted significance threshold ($P < 2.24 \times 10^{-10}$).

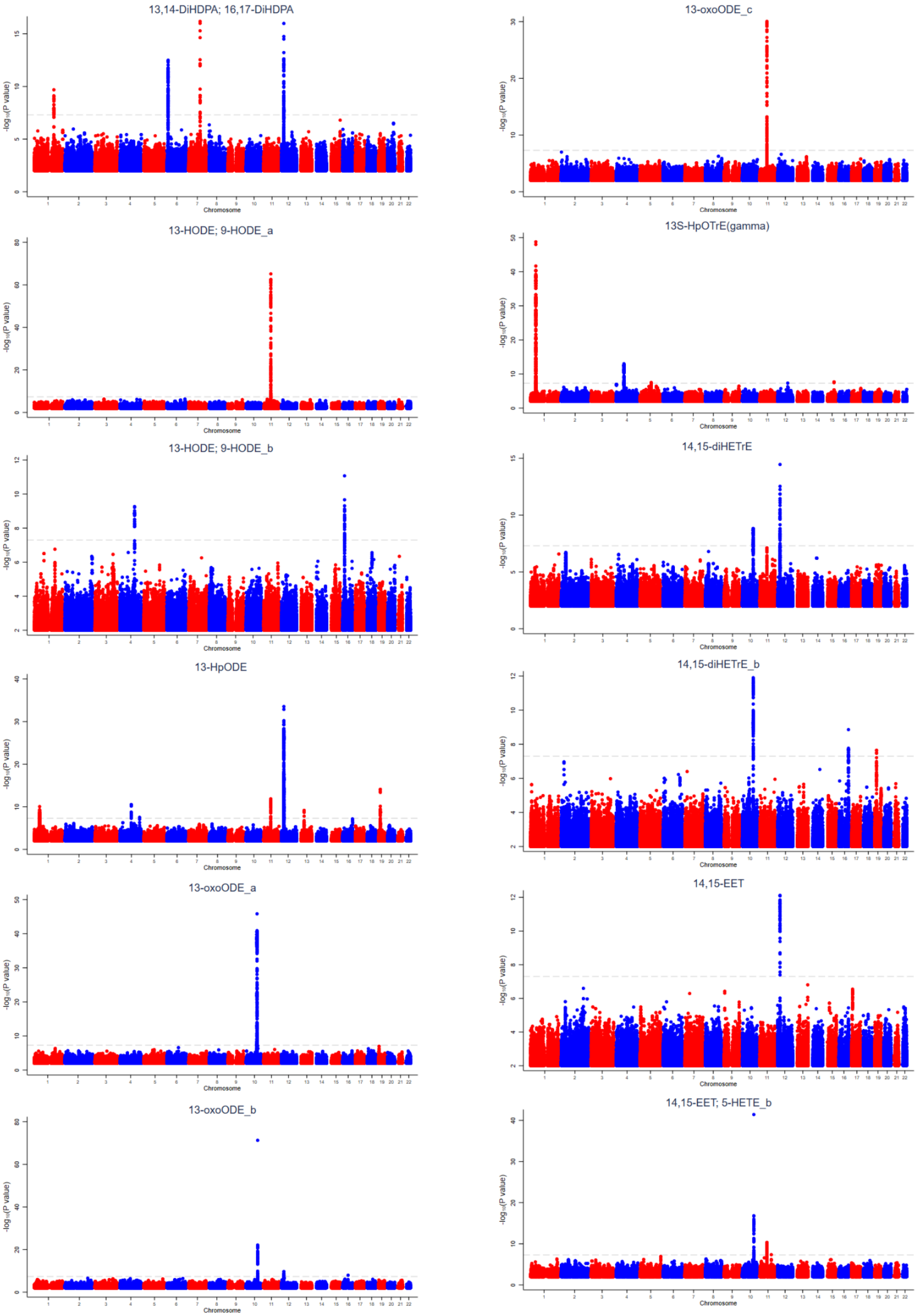

Supplementary Figure 4. Significant genome-wide eicosanoid associations (25-36). Manhattan plots for eicosanoids with at least one significant association, with association signals (-log10 of P-value) on the y-axis versus SNPs according to their position in the genome on the x-axis. Horizontal dashed lines represent Bonferroni adjusted significance threshold ($P < 2.24 \times 10^{-10}$).

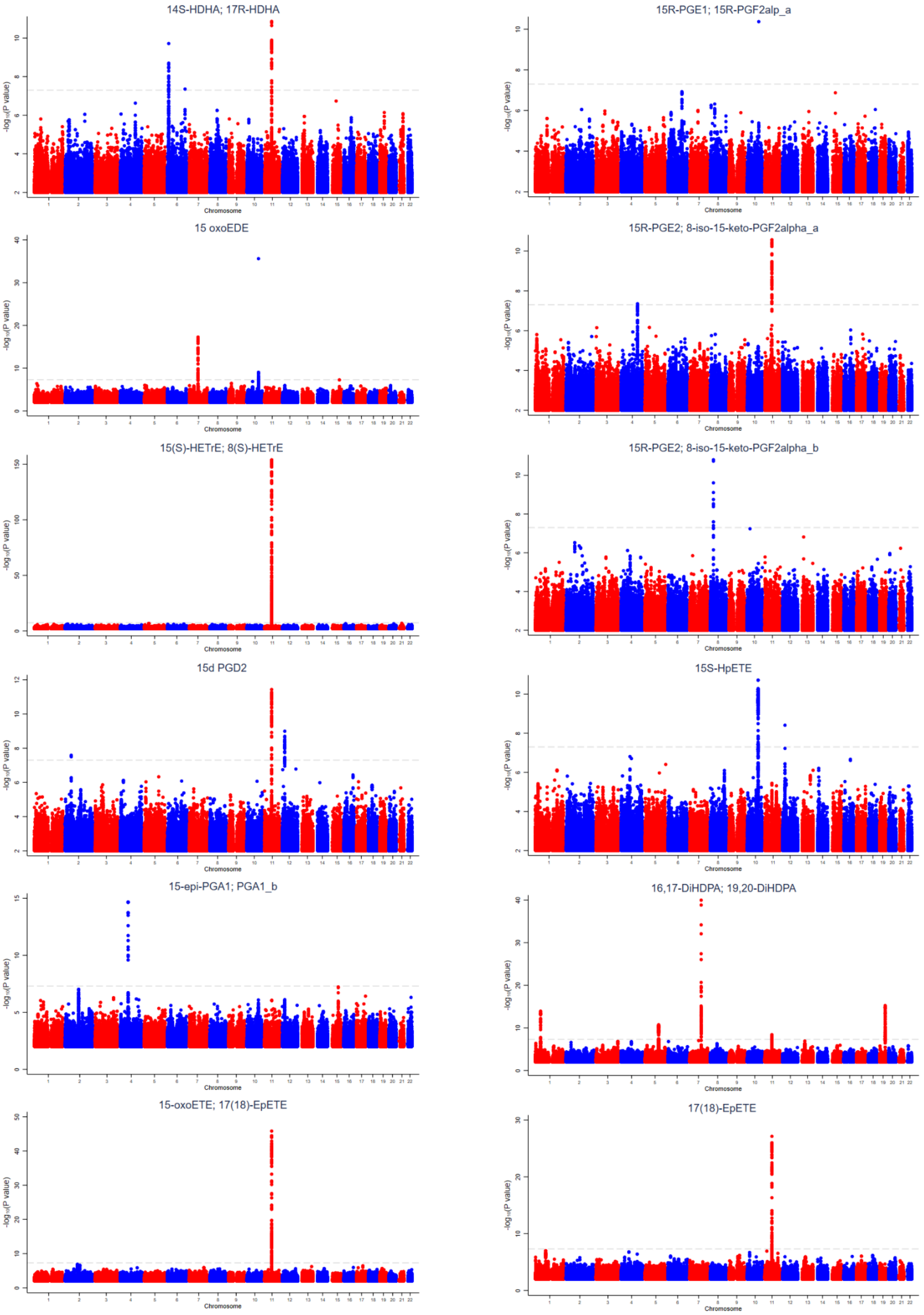

Supplementary Figure 5. Significant genome-wide eicosanoid associations (37-48). Manhattan plots for eicosanoids with at least one significant association, with association signals (-log10 of P-value) on the y-axis versus SNPs according to their position in the genome on the x-axis. Horizontal dashed lines represent Bonferroni adjusted significance threshold ($P < 2.24 \times 10^{-10}$).

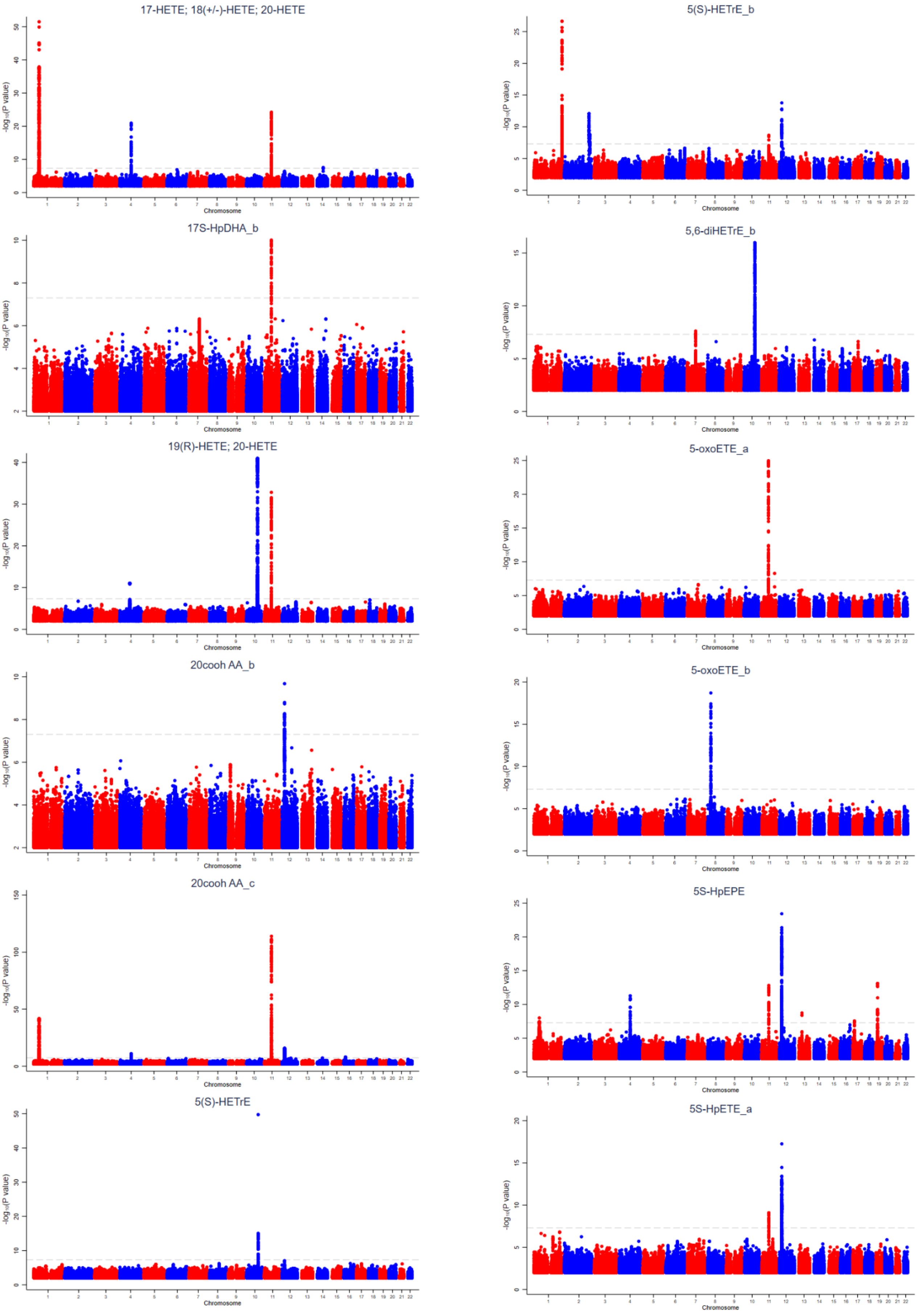

Supplementary Figure 6. Significant genome-wide eicosanoid associations (49-60). Manhattan plots for eicosanoids with at least one significant association, with association signals (-log10 of P-value) on the y-axis versus SNPs according to their position in the genome on the x-axis. Horizontal dashed lines represent Bonferroni adjusted significance threshold ($P < 2.24 \times 10^{-10}$).

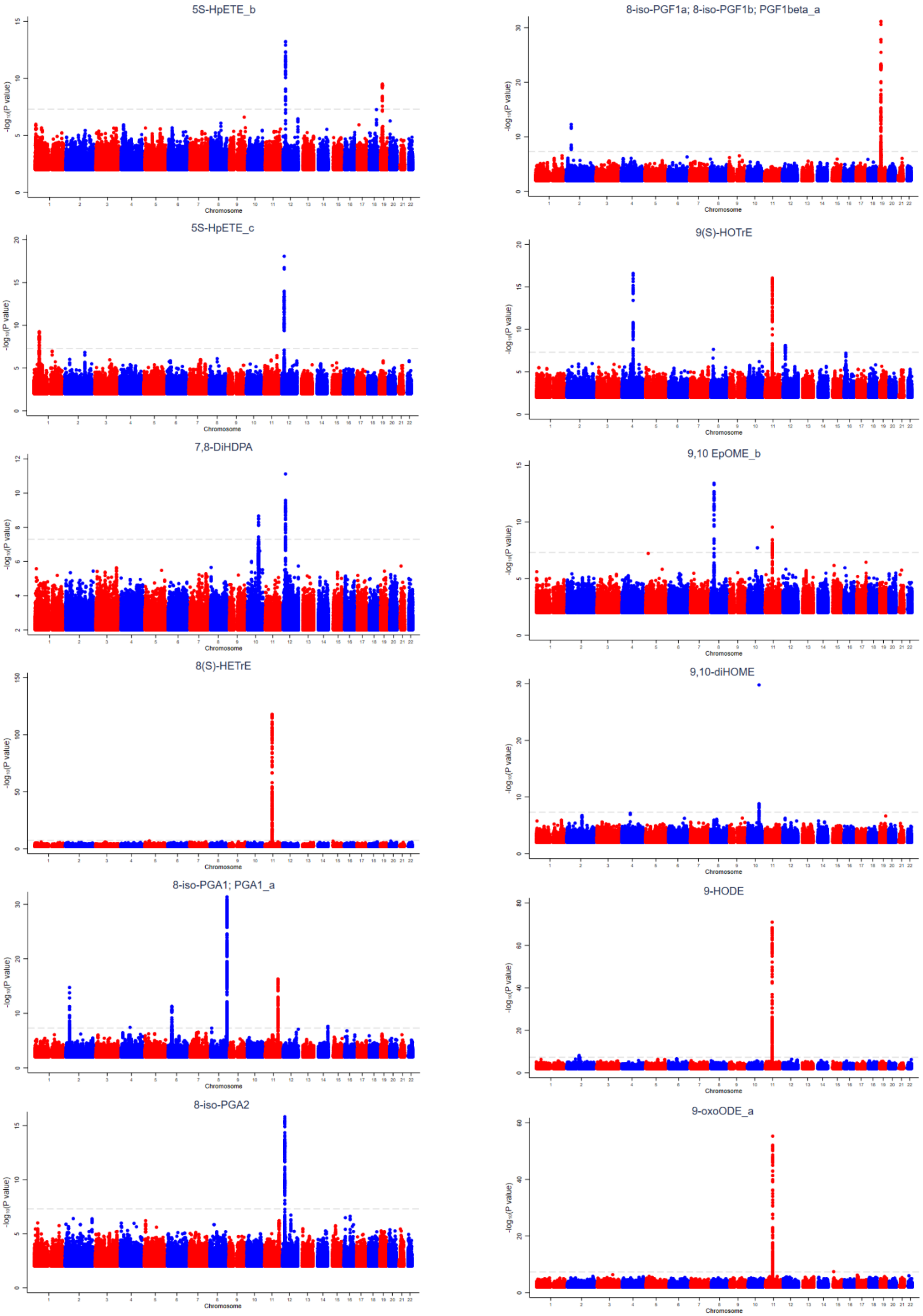

Supplementary Figure 7. Significant genome-wide eicosanoid associations (61-72). Manhattan plots for eicosanoids with at least one significant association, with association signals (-log10 of P-value) on the y-axis versus SNPs according to their position in the genome on the x-axis. Horizontal dashed lines represent Bonferroni adjusted significance threshold ($P < 2.24 \times 10^{-10}$).

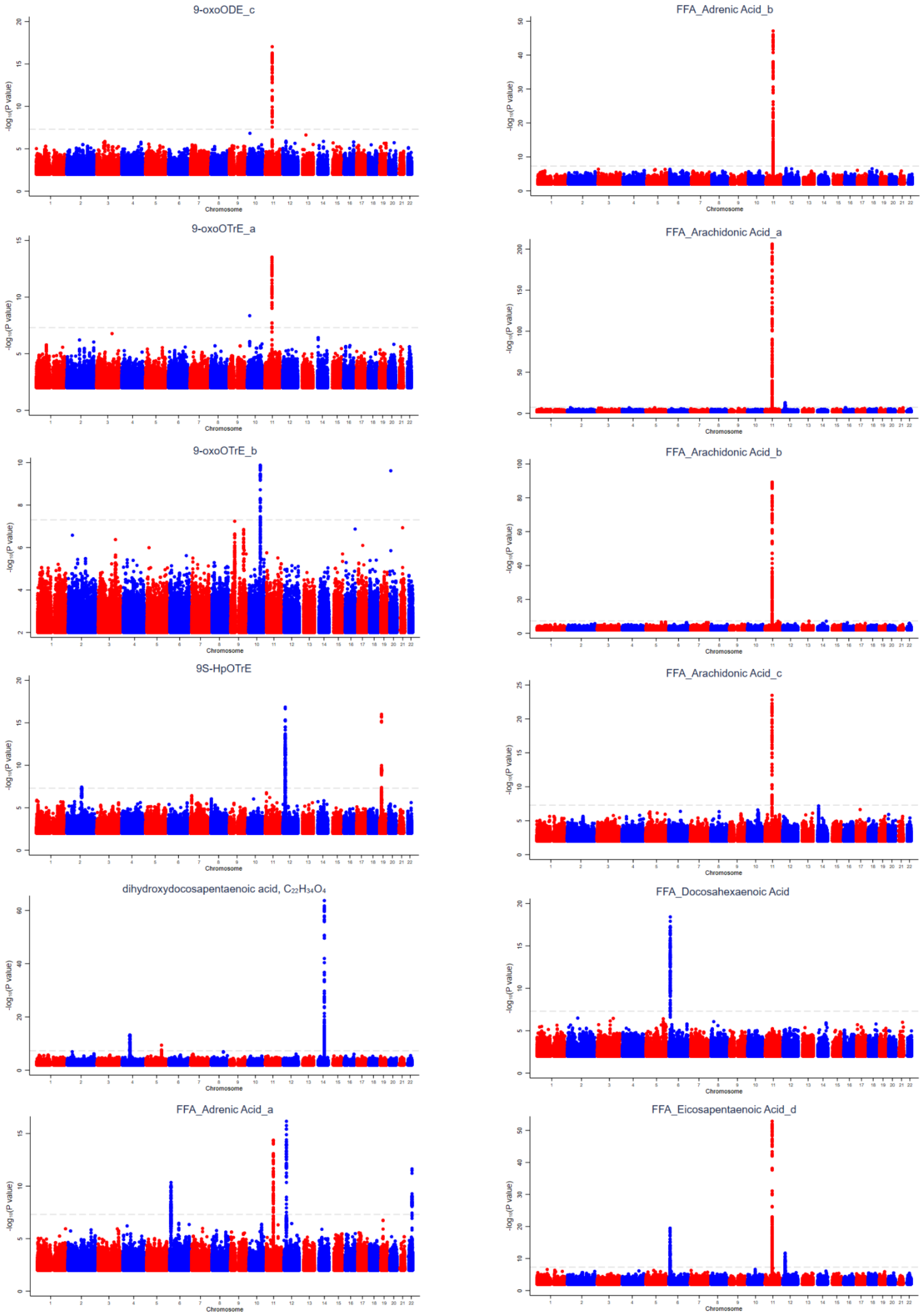

Supplementary Figure 8. Significant genome-wide eicosanoid associations (73-84). Manhattan plots for eicosanoids with at least one significant association, with association signals (-log10 of P-value) on the y-axis versus SNPs according to their position in the genome on the x-axis. Horizontal dashed lines represent Bonferroni adjusted significance threshold ($P < 2.24 \times 10^{-10}$).

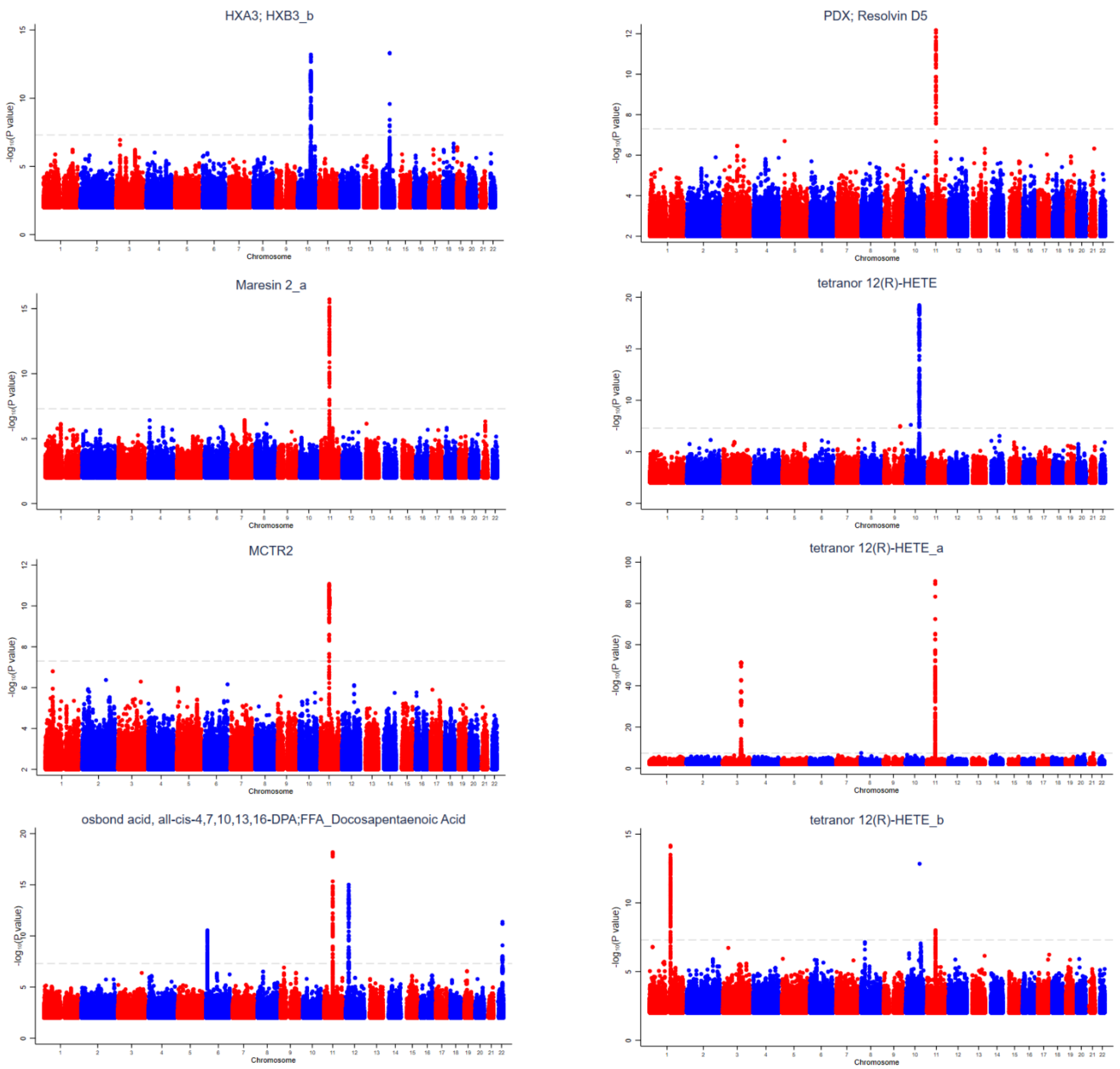

Supplementary Figure 9. Significant genome-wide eicosanoid associations (85-92). Manhattan plots for eicosanoids with at least one significant association, with association signals (-log10 of P-value) on the y-axis versus SNPs according to their position in the genome on the x-axis. Horizontal dashed lines represent Bonferroni adjusted significance threshold (P < 2.24 x 10^{-10}).

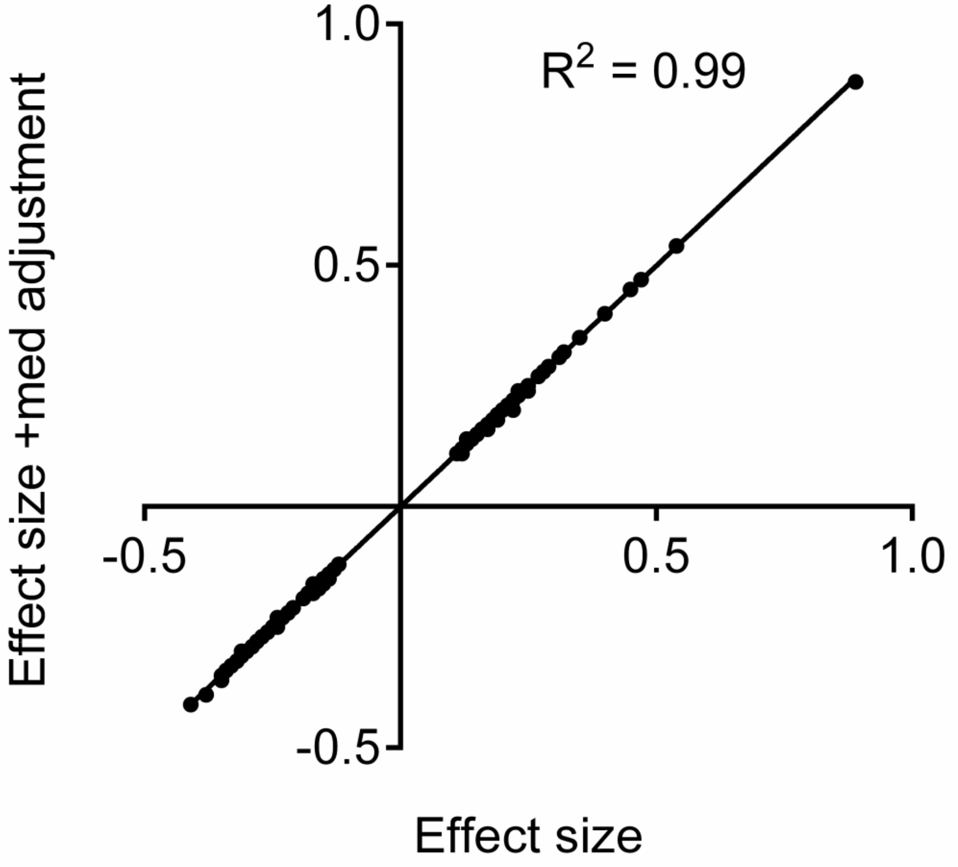

Supplementary Figure 10. Comparison of GWAS with or without medication adjustment. Scatter plot of effect sizes of significant loci in main GWAS analysis (x-axis) versus effect sizes in GWAS further adjusted for aspirin and NSAID use (y-axis).
