# Supplementary material for: A genome-wide association study identifies 41 loci associated with eicosanoid levels
Source: Commun Biol. 2023 Jul 31;6:792. doi: 10.1038/s42003-023-05159-5 (PMC10390489; doi:10.1038/s42003-023-05159-5)
Supplement: Supplementary file 3 — Description of Additional Supplementary Files [file 42003_2023_5159_MOESM3_ESM.pdf]

## Description of Additional Supplementary Files

**File Name:** Supplementary Data 1
**Description:** Eicosanoids and related metabolites measured in ARIC.

**File Name:** Supplementary Data 2
**Description:** Significant GWAS associations (P<2.24 x 10-10).

**File Name:** Supplementary Data 3
**Description:** SNPs within 500 Mb of *PTGS1* and *PTGS2*.

**File Name:** Supplementary Data 4
**Description:** TWAS for top SNPs associated with eicosanoids (P<0.0012).
