# Supplementary material for: A genome-wide association study identifies 41 loci associated with eicosanoid levels
Source: Commun Biol. 2023 Jul 31;6:792. doi: 10.1038/s42003-023-05159-5 (PMC10390489; doi:10.1038/s42003-023-05159-5)
Supplement: Supplementary file 5 — Reporting Summary [file 42003_2023_5159_MOESM5_ESM.pdf]

nature portfolio

Corresponding author(s): Eugene Rhee

Last updated by author(s): Jul 6, 2023

Reporting Summary

Nature Portfolio wishes to improve the reproducibility of the work that we publish. This form provides structure for consistency and transparency in reporting. For further information on Nature Portfolio policies, see our Editorial Policies and the Editorial Policy Checklist.

Statistics

For all statistical analyses, confirm that the following items are present in the figure legend, table legend, main text, or Methods section.

n/a	Confirmed	
☒	☐	The exact sample size (*n*) for each experimental group/condition, given as a discrete number and unit of measurement
☒	☐	A statement on whether measurements were taken from distinct samples or whether the same sample was measured repeatedly
☐	☒	The statistical test(s) used AND whether they are one- or two-sided<br>*Only common tests should be described solely by name; describe more complex techniques in the Methods section.*
☐	☒	A description of all covariates tested
☐	☒	A description of any assumptions or corrections, such as tests of normality and adjustment for multiple comparisons
☐	☒	A full description of the statistical parameters including central tendency (e.g. means) or other basic estimates (e.g. regression coefficient) AND variation (e.g. standard deviation) or associated estimates of uncertainty (e.g. confidence intervals)
☐	☒	For null hypothesis testing, the test statistic (e.g. *F*, *t*, *r*) with confidence intervals, effect sizes, degrees of freedom and *P* value noted<br>*Give P values as exact values whenever suitable.*
☒	☐	For Bayesian analysis, information on the choice of priors and Markov chain Monte Carlo settings
☒	☐	For hierarchical and complex designs, identification of the appropriate level for tests and full reporting of outcomes
☐	☒	Estimates of effect sizes (e.g. Cohen's *d*, Pearson's *r*), indicating how they were calculated

Our web collection on statistics for biologists contains articles on many of the points above.

Software and code

Policy information about availability of computer code

Data collection	No software was used
Data analysis	The Trans-Omics for Precision Medicine reference (Freeze 5b) was used for data imputation. GWAS was conducted using Fast Association Tests (FAST) software. Index SNPs were annotated through linkage with the SNiPA web-tool based on the 1000 Genomes phase 3 v5 and Ensembl v87 datasets. We performed transcriptome-wide association studies (TWAS) using models from the GTEx project v8 (http://gusevlab.org/projects/fusion/)

For manuscripts utilizing custom algorithms or software that are central to the research but not yet described in published literature, software must be made available to editors and reviewers. We strongly encourage code deposition in a community repository (e.g. GitHub). See the Nature Portfolio guidelines for submitting code & software for further information.

Data

Policy information about availability of data

All manuscripts must include a data availability statement. This statement should provide the following information, where applicable:

- Accession codes, unique identifiers, or web links for publicly available datasets
- A description of any restrictions on data availability
- For clinical datasets or third party data, please ensure that the statement adheres to our policy

Summary level data will be made available at the NHGRI-EBI catalog (https://www.ebi.ac.uk/gwas/home). The informed consents given by ARIC study participants

do not cover data posting in public databases. However, data are available upon request from ARIC (https://sites.cscc.unc.edu/aric/contact_the_coord_center). Data requests can be submitted online and are subject to approval by the ARIC Steering Board.

Human research participants

Policy information about studies involving human research participants and Sex and Gender in Research.

Reporting on sex and gender	The ARIC study sample was comprised of 4647 females and 3759 males. Sex- and gender-based analyses were not conducted because they are not expected to impact how genetic variants impact blood eicosanoid levels.
Population characteristics	The Atherosclerosis Risk in Communities (ARIC) Study is a community-based prospective cohort study. For this study, the total sample included 8406 individuals, with mean age 56.9, 55.3% female, 26.6% with hypertension, and 14.1% with diabetes; 6496 were of European ancestry and 1910 of African ancestry.
Recruitment	Study participants were enrolled from Forsyth County, North Carolina, Jackson, Mississippi, suburbs of Minneapolis, Minnesota, and Washington County, Maryland from 1987-1989. Formal written consent was obtained.
Ethics oversight	Research was approved by Johns Hopkins IRB00012998 with data repository procedures approved by IRB00311861 for the preparation and analysis of ARIC research datasets.

Note that full information on the approval of the study protocol must also be provided in the manuscript.

Field-specific reporting

Please select the one below that is the best fit for your research. If you are not sure, read the appropriate sections before making your selection.

☒ Life sciences ☐ Behavioural & social sciences ☐ Ecological, evolutionary & environmental sciences

For a reference copy of the document with all sections, see nature.com/documents/nr-reporting-summary-flat.pdf

Life sciences study design

All studies must disclose on these points even when the disclosure is negative.

Sample size	No sample size collection. All individuals in the ARIC study who had undergone both genotyping and plasma eicosanoid profiling were included, yielding a total of 8406 individuals.
Data exclusions	Exclusion criteria were pre-established. Eicosanoids missing in more than 50% of the samples were dropped, leaving 223 eicosanoids for statistical analysis
Replication	Meta-analysis was conducted across European and African ancestry cohorts.
Randomization	Not relevant, as no distinct experimental groups were not assigned for GWAS.
Blinding	Not relevant, as no distinct experimental groups were not assigned for GWAS.

Reporting for specific materials, systems and methods

We require information from authors about some types of materials, experimental systems and methods used in many studies. Here, indicate whether each material, system or method listed is relevant to your study. If you are not sure if a list item applies to your research, read the appropriate section before selecting a response.

Materials & experimental systems

n/a	Involved in the study	
☒	☐	Antibodies
☒	☐	Eukaryotic cell lines
☒	☐	Palaeontology and archaeology
☒	☐	Animals and other organisms
☐	☒	Clinical data
☒	☐	Dual use research of concern

Methods

n/a	Involved in the study	
☒	☐	ChIP-seq
☒	☐	Flow cytometry
☒	☐	MRI-based neuroimaging

Clinical data

Policy information about clinical studies

All manuscripts should comply with the ICMJE guidelines for publication of clinical research and a completed CONSORT checklist must be included with all submissions.

Clinical trial registration	N/A
Study protocol	N/A, not a clinical trial
Data collection	Study participants were enrolled from Forsyth County, North Carolina, Jackson, Mississippi, suburbs of Minneapolis, Minnesota, and Washington County, Maryland from 1987-1989.
Outcomes	Primary outcome was plasma eicosanoid levels measured by LC-MS
